# Inhalational Chlorine Injuries at Public Aquatic Venues — California, 2008–2015

**DOI:** 10.15585/mmwr.mm6619a3

**Published:** 2017-05-19

**Authors:** Jason A. Wilken, Michele DiMaggio, Matthew Kaufmann, Kevin O’Connor, Svetlana Smorodinsky, Christina Armatas, Tracy Barreau, Richard Kreutzer, Lino Ancheta

**Affiliations:** ^1^Division of Environmental and Occupational Disease Control, California Department of Public Health, Richmond, California; ^2^Career Epidemiology Field Officer Program, Field Services Branch, Division of State and Local Readiness, Office of Public Health Preparedness and Response, CDC; ^3^Contra Costa Environmental Health, Martinez, California; ^4^Hazardous Materials Division, Contra Costa Health Services, Martinez, California.

In June 2015, personnel from California’s Contra Costa Health Services Environmental Health and Hazardous Materials (hazmat) divisions were alerted to a possible chemical release at a swimming pool in an outdoor municipal water park. Approximately 50 bathers were in the pool when symptoms began; 34 (68%) experienced vomiting, coughing, or eye irritation. Among these persons, 17 (50%) were treated at the scene by Contra Costa’s Emergency Medical Services (EMS) and released, and 17 (50%) were transported to local emergency departments; five patients also were evaluated later at an emergency department or by a primary medical provider. Environmental staff members determined that a chemical controller malfunction had allowed sodium hypochlorite and muriatic acid (hydrochloric acid) solutions to be injected into the main pool recirculation line while the recirculation pump was off; when the main recirculation pump was restarted, toxic chlorine gas (generated by the reaction of concentrated sodium hypochlorite and muriatic acid) was released into the pool. A review of 2008–2015 California pesticide exposure records identified eight additional such instances of toxic chlorine gas releases at public aquatic venues caused by equipment failure or human error that sickened 156 persons. Chemical exposures at public aquatic venues can be prevented by proper handling, storage, and monitoring of pool chemicals; appropriate equipment operation and maintenance; training of pool operators and staff members on pool chemical safety; and reporting of chemical exposures.

On June 18, 2015, at 2:29 p.m., an initial 9-1-1 call reported 10–12 persons experiencing vomiting or respiratory symptoms at one of five swimming pools at an outdoor municipal water park in Contra Costa County. Contra Costa EMS and fire department personnel were dispatched. At 2:42 p.m., fire personnel requested that hazmat personnel assist in incident response, but at 2:44 p.m., the request was cancelled after fire personnel determined that there was no active chemical leak. At 3:07 p.m., fire personnel again requested hazmat personnel to investigate a possible chemical leak. Hazmat staff members arrived at the water park at 4:14 p.m. and Environmental Health personnel arrived at 4:30 p.m.; both integrated into a fire department–led incident command structure. EMS personnel evaluated and transported patients to local emergency departments. Among the 17 patients transported to an emergency department, 16 (94%) were released the same day; one patient who was experiencing tachycardia and wheezing was admitted for monitoring and breathing treatments and discharged the next day. Hazmat staff members reviewed the pool chemical controller data and performed air monitoring around the perimeter of the water park, within the immediate vicinity of the affected pool, and at the chemical storage building. Environmental Health staff members measured the free chlorine concentrations and pH of each pool and interviewed municipal water park employees and the pool maintenance contractor.

Hazmat personnel did not detect chlorine in the air during sampling conducted >2 hours after the initial 9-1-1 call. The free chlorine concentration measured in the water of the affected pool >2 hours after the initial 9-1-1 call was 10.5–13.5 ppm, and the pH was 6.8. Both measurements were in violation of California regulations, which allow a maximum of 10 ppm free chlorine and a pH range of 7.2–7.8 (*1*). The free chlorine concentrations also violated the manufacturer’s label instructions that allow a concentration of no greater than 4 ppm in pool water. The pH of the water in two of the other four pools at the park was also <7.0. Environmental Health ordered that the water park and the pool where the exposure incident occurred be immediately closed and remain closed until Environmental Health completed a review of the park’s remediation plan for the pool (*2*).

During normal operations, pool water was drawn from the pool by a recirculation pump. A chemical controller regulated feed of muriatic acid and sodium hypochlorite solutions into the recirculation line, diluting these chemicals and allowing them to mix safely. (Sodium hypochlorite provides the chlorine necessary to inactivate infectious pathogens, and muriatic acid maintains the pool pH within a range that maximizes the chlorine’s effectiveness.) The chemical controller was equipped with a rotary flow sensor, interlocked with an accompanying overfeed alarm to prevent chemical feed in the absence of recirculation flow. Review of the chemical controller data identified a recorded zero flow rate of the main recirculation pump (Figure) for approximately 16 hours beginning at 10:40 p.m. on June 17, the day before the incident. During these 16 hours leading up to the incident, the chemical controller also recorded intermittent chemical dispensing of sodium hypochlorite (approximately 81 gallons over a total period of 218 minutes) and muriatic acid (approximately 2 gallons over a total period of 39 minutes) into the recirculation line in the absence of recirculation flow caused by an unknown equipment failure. This allowed these concentrated chemicals to mix and generate toxic chlorine gas. At approximately 8:40 a.m., for unknown reasons, the over-feed alarm was turned off by aquatic staff members. At about 2:20 p.m., aquatic staff members turned on the main recirculation pump, and approximately 10 minutes later EMS received a 9-1-1 call from water park employees.

**FIGURE:**
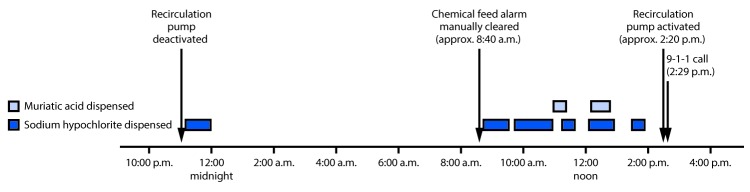
Timeline of events showing deactivation and reactivation of recirculation pump, release of sodium hypochlorite and muriatic acid into the pool water,* and call to emergency services — Municipal Water Park, Contra Costa County, California, June 17–18, 2015 * Caused by an unknown equipment failure

To characterize such chemical exposures at public aquatic facilities, investigators reviewed California Department of Pesticide Regulation (CDPR) Pesticide Episode Notification Record* extracts recorded during 2008–2015. Eight additional toxic chlorine releases with multiple persons injured by each release were identified, and Pesticide Episode Closing Reports of each of these incidents were reviewed. Medical records were obtained for nine incidents occurring in 2015 (Table). Among all nine incidents, a total of 155 persons (median = 16 per incident, range = 2–34) were symptomatic (primarily respiratory symptoms, vomiting, and eye irritation), 121 (78%) patients were transported to an emergency department or were evaluated by their primary medical provider (median = 11 per incident, range = 2–27), and five of 70 (7%) persons for whom information on hospitalization was available required hospital admission (median = 1 per incident, range = 0–2). Factors contributing to these incidents included one or more of the following: chemical controller failures, valve failures, or human error. A feature noted in all events was that chemicals were inappropriately dispensed while main recirculation pumps were deactivated, causing the chemicals to mix at concentrations that resulted in the generation of toxic chlorine gas. In seven of the nine incidents, main recirculation pumps were turned on while bathers were present in pools.

**TABLE:** Chorine gas exposure incidents at public aquatic venues — California, 2008–2015

Date	County	Pool location/type	No. persons			Identified cause
			Symptomatic	Evaluated at ED/PMP	Admitted to hospital ≥24 hours	
Oct 2008	Orange	High school swimming	6	6	0	Chemical metering device failed; recirculation pump restarted while bathers in pool
Jun 2009	Los Angeles	Municipal	7	7	0	Chemical valves not closed during replacement of recirculation pump; recirculation pump replaced and activated while bathers in pool
Jul 2010	Los Angeles	Municipal	30*	17	—^†^	Attempted to prime a dry chemical line while bathers in pool
Aug 2010	San Mateo	Municipal	2	2	1	Unknown
Nov 2010	Santa Clara	Privately owned	19	11	—^†^	Flow switch monitor failure; recirculation pump restarted while bathers in pool
Aug 2011	Sacramento	Privately owned	24	24	—^†^	Chemical controller manually bypassed; recirculation pump restarted while bathers in pool
Jun 2015	Shasta	Privately owned fitness facility	28	27	2	Chemical valve failure; recirculation pump restarted while bathers in pool
Jun 2015	Contra Costa	Municipal	34	22	1	Chemical controller failure and chemical controller manually bypassed; recirculation pump restarted while bathers in the pool
Oct 2015	Contra Costa	High school swimming	5	5	1	Chemical controller failure; recirculation pump restarted while bathers in the pool

**Abbreviations:** ED = emergency department; PMP = primary medical provider.

* Approximate number.

^†^ California Department of Pesticide Regulation records did not indicate whether patients were admitted to a hospital.

## Discussion

An estimated >50 million persons swim for sport or recreation in the United States each year (*3*). Proper management of chemical disinfection of pool water is essential to prevent transmission of infectious pathogens. In 2012, an estimated 4,876 visits to emergency departments occurred after pool chemical–associated health events, such as those highlighted in this report (*4*).

Compliance with California and by Contra Costa County regulations might have prevented the incident described in this report. In addition to regulations defining limits for free chlorine concentrations and pH in pool water, the California Code of Regulations also requires that chemical feeder equipment shall “be maintained and repaired according to manufacturer’s specifications” (*5*). The California Code of Regulations also requires the recirculation pump to be in operation whenever the public pool is available for use (*6*). However, no federal agencies regulate public aquatic facility design, construction, operation, and maintenance; rather, these regulations are written, enacted, implemented, and enforced by state and local jurisdictions. CDC and the New York Department of Health have spearheaded development of the Model Aquatic Health Code (MAHC; https://www.cdc.gov/mahc/editions/current.html), a science- and best practices–based resource for preventing public aquatic facility–associated illness and injury. MAHC development and maintenance is the result of an ongoing collaboration among federal, state, and local public health officials, and representatives from the aquatics sector. MAHC is updated biennially (https://www.cmahc.org/), most recently in 2016. State or local jurisdictions can voluntarily use MAHC sections as a resource and a guide to prevent illness and injury associated with public aquatic venues. MAHC section 5.7.3.5.1.3 (Fail Proof Safety Features) states that equipment must be unable to feed chemicals in the absence of recirculation flow, and MAHC section 4.7.3.2.1.3 (Interlock Controls and No or Low Flow Deactivation) describes criteria for automatic shutoff of chemical feeders by an interlock in the case of interruption of recirculation flow. In the 2015 incident described in this report, according to the manufacturer’s specifications, the chemical feeder interlock should have shut off all chemical feeding while the recirculation pump was off but an unknown equipment failure allowed pool chemicals to be delivered intermittently during a 16-hour period. In addition, for unknown reasons members of the aquatic staff turned off the alarm.

The findings in this report are subject to at least three limitations. First, chlorine concentrations in the water and air can fluctuate, and sampling >2 hours after the incident likely did not reflect concentrations at the time the symptoms occurred. Second, in the retrospective analysis, only those incidents reported to CDPR could be identified; other incidents might have occurred and were not reported to CDPR. Finally, medical records associated with incidents before 2015 were not obtained for this analysis.

Despite these limitations, this investigation revealed that the public health impact of toxic chlorine gas releases might be reduced or mitigated by following practices recommended in MAHC, and the incident described above might have been prevented by the additional following steps. Public aquatic facilities can perform regular challenge tests of chemical feeder interlock systems (e.g., regular measurement of time for the interlock to shut off chemical feeders after the recirculation pump turns off), and conduct these tests when no bathers are in the pool. An audible or visual alarm system can be incorporated to indicate when the recirculation pump is off. It is important that all bathers be evacuated from the aquatic venue if the recirculation pump is off (regardless of reason) or when chemical feeders are deactivated, and that they not be allowed to reenter the aquatic venue until the cause of recirculation pump deactivation has been identified and corrected. Furthermore, it is important that bathers not be allowed to reenter the aquatic venue until water quality measurements return to concentrations allowed by standards. Aquatic staff members can be trained to recognize the signs and symptoms of chlorine exposure and how to respond if noted. Public aquatic venues can develop, train, and test emergency action plans including notification of all applicable local regulatory agencies and emergency response teams. Hazardous materials and environmental health personnel can be promptly integrated into responses to potential chemical exposures at public aquatic venues to identify contributing factors, which can be addressed in future prevention efforts.

**Summary**

**What is already known about this topic?**

Equipment failure and human error at public aquatic venues can lead to toxic chlorine gas releases and have negative health impacts on bathers and aquatic staff members.

**What is added by this report?**

A multiagency investigation identified both equipment failure and human error as root causes of a toxic chlorine gas release and resulting exposures at a Contra Costa County, California municipal water park. A review of 2008–2015 California Department of Pesticide Regulation records identified contributing factors of toxic chlorine gas exposures at public aquatic venues, including equipment failure, human error, and restarting of a recirculation pump while bathers were present in pools.

**What are the implications for public health practice?**

Toxic chlorine gas releases at public aquatic venues can be prevented by regular testing of chemical control failsafe features, proper training of aquatic facility staff members, and by following standardized policies and procedures, including evacuating bathers from the pool before a recirculation pump is restarted. State or local jurisdictions can voluntarily use CDC’s Model Aquatic Health Code (https://www.cdc.gov/mahc/editions/current.html) as a resource and guide of standardized, evidence-based regulations designed to prevent injuries and illness at public aquatic venues.

## Footnotes

* Pesticide Episode Notification Records are California Department of Pesticide Regulation (CDPR) enforcement forms completed by staff members at CDPR’s Enforcement Regional Office, which provides oversight to counties when they are either notified by the county of an exposure incident or by the CDPR Pesticide Illness Surveillance Program of an exposure incident that meets the priority status as defined by a cooperative agreement with Environmental Protection Agency Region 9.
